# Waning antibodies from inactivated SARS-CoV-2 vaccination offer protection against infection without antibody-enhanced immunopathology in rhesus macaque pneumonia models

**DOI:** 10.1080/22221751.2021.2002670

**Published:** 2021-11-21

**Authors:** Dandan Li, Ning Luan, Jing Li, Heng Zhao, Ying Zhang, Runxiang Long, Guorun Jiang, Shengtao Fan, Xingli Xu, Han Cao, Yunfei Wang, Yun Liao, Lichun Wang, Longding Liu, Cunbao Liu, Qihan Li

**Affiliations:** Yunnan Key Laboratory of Vaccine Research and Development on Severe Infectious Diseases, Institute of Medical Biology, Chinese Academy of Medical Sciences and Peking Union Medical College, Kunming, People’s Republic of China

**Keywords:** SARS-CoV-2, ADE, subneutralizing antibodies, inactivated vaccines, immunopathology

## Abstract

Inactivated coronaviruses, including severe acute respiratory syndrome coronavirus 1 (SARS-CoV-1) and Middle East respiratory syndrome coronavirus (MERS-CoV), as potential vaccines have been reported to result in enhanced respiratory diseases (ERDs) in murine and nonhuman primate (NHP) pneumonia models after virus challenge, which poses great safety concerns of antibody-dependent enhancement (ADE) for the rapid wide application of inactivated SARS-CoV-2 vaccines in humans, especially when the neutralizing antibody levels induced by vaccination or initial infection quickly wane to nonneutralizing or subneutralizing levels over the time. With passive transfer of diluted postvaccination polyclonal antibodies to mimic the waning antibody responses after vaccination, we found that in the absence of cellular immunity, passive infusion of subneutralizing or nonneutralizing anti-SARS-CoV-2 antibodies could still provide some level of protection against infection upon challenge, and no low-level antibody-enhanced infection was observed. The anti-SARS-CoV-2 IgG-infused group and control group showed similar, mild to moderate pulmonary immunopathology during the acute phase of virus infection, and no evidence of vaccine-related pulmonary immunopathology enhancement was found. Typical immunopathology included elevated MCP-1, IL-8 and IL-33 in bronchoalveolar lavage fluid; alveolar epithelial hyperplasia; and exfoliated cells and mucus in bronchioles. Our results corresponded with the recent observations that no pulmonary immunology was detected in preclinical studies of inactivated SARS-CoV-2 vaccines in either murine or NHP pneumonia models or in large clinical trials and further supported the safety of inactivated SARS-CoV-2 vaccines.

Antibody-dependent enhancement (ADE) effect, defined as the antibodies induced by initial infection or vaccination binding to viral surface proteins and promote viral invasion of host cells, is a major concern for all vaccine developers. Inactivated coronaviruses, including severe acute respiratory syndrome coronavirus (SARS-CoV) and Middle East respiratory syndrome coronavirus (MERS-CoV), as potential vaccines have been reported to result in enhanced respiratory diseases (ERDs) in murine and nonhuman primate (NHP) pneumonia models after virus challenge, which poses great safety concerns for the rapid wide application of inactivated severe acute respiratory syndrome coronavirus 2 (SARS-CoV-2) vaccines in humans [1,2]. No ERD typical of an increased proinflammatory pulmonary response upon challenge was detected in preclinical studies of inactivated SARS-CoV-2 vaccines in either murine or NHP pneumonia models or in large clinical trials[3,4]. However, with the neutralizing antibody response induced by vaccination or initial infection weakening over time [5,6], whether nonneutralizing or subneutralizing antibodies would lead to enhanced viral invasion or proinflammatory damage upon reinfection is becoming a serious concern. Using passive transfer of diluted postvaccination polyclonal antibodies to mimic the waning antibody responses after vaccination, ADE has been reported both in vitro and in vivo for SARS-CoV and MERS-CoV [7,8]. To estimate the risk of ERD caused by decreased neutralizing antibody levels after vaccination with inactivated SARS-CoV-2, we adopted a similar strategy to study potential vaccine-associated enhanced respiratory disease (VAERD) in well-established rhesus macaque pneumonia models [9].

Anti-serum with a neutralizing antibody geometric mean titre (GMT) > 1:128 was collected from rhesus macaque intramuscularly injected on days 0 and 28 with a formaldehyde and β-propiolactone double-inactivated SARS-CoV-2 vaccine developed by the Institute of Medical Biology, Chinese Academy of Medical Sciences (IMBCAMS) based on the KMS-1 strain (GenBank accession number MT226610.1) [10]. Anti-SARS-CoV-2 IgG was purified from the anti-serum using MabSelect affinity resin (GE Healthcare Bio-Sciences AB, Sweden), while IgG from rhesus monkeys immunized with an inactivated EV-71 vaccine developed by IMBCAMS was purified and used as a control [11,12]. Six rhesus macaques (male, age 1.5 years) that were randomly divided into two groups (4 for the anti-SARS-CoV-2 IgG group and 2 for the control IgG group) were injected intravenously with purified IgG at a sub-neutralizing or non-neutralizing dose of 10 mg/kg body weight (Figure 1a). Three days after IgG injection, all animals were infected with SARS-CoV-2 via a bilateral nasal drip at a dose of 10^5^ TCID50. Samples, including nasal swabs, pharyngeal swabs and blood samples, were collected daily for viral shedding and neutralizing titre analyses. Four days after SARS-CoV-2 infection, all animals were sacrificed after anesthesia. Bronchoalveolar lavage fluid (BALF) and the main tissues and organs were collected for proinflammatory mediator, viral load and histopathology analyses.

**Figure 1.**
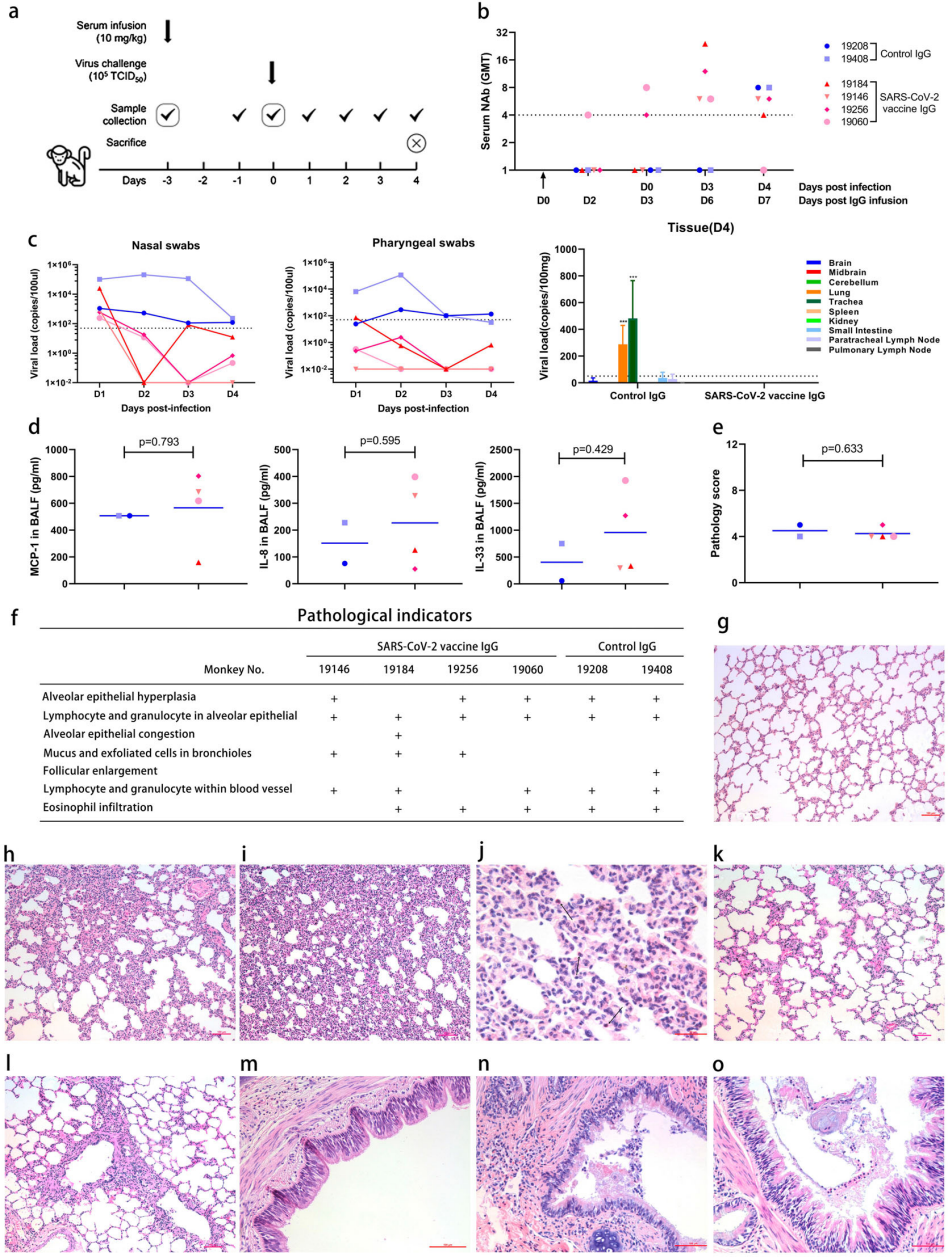
Protection against infection upon SARS-CoV-2 challenge provided by waning antibodies from inactivated SARS-CoV-2 vaccination in rhesus macaques. (a) Scheme of the experimental design. (b) Serum neutralizing antibody (NAb) geometric mean titres (GMTs) before and after virus challenge. Each point represents one animal. A titre of neutralizing antibodies less than 1:4 was designated negative (value = 1) in the GMT calculation. (c) Viral loads at the indicated time points (for nasal swabs and pharyngeal swabs, each line represents one animal) and on sacrifice day (for different tissues, the results for each group are presented as means ± SDs). A viral copy number of less than 50copies/100μl or 50copies/100 mg (dotted lines) was considered negative. (d) Proinflammatory mediator concentrations in bronchoalveolar lavage fluid (BALF) on the sacrifice day. Duplicate wells were performed in all experiments, each point represents average value for duplicate wells. (e) Pathology score. The average estimated severity of the lesions was determined by two pathologists in at least five areas at 100x as well as 200× magnification and scored as 1 (weak), 2 (moderate) or 3 (severe); and the total score was calculated by adding the scores of the left & right lung and superior & inferior lobe of each animal. Each point represents one animal. (f) Pathological indicators of each animal lung tissue with H&E staining. (g) Normal alveoli. (h) Mild alveolar epithelial hyperplasia. (i) Moderate alveolar epithelial hyperplasia. (j) Eosinophil infiltration indicated by arrows. (k) Alveolar epithelial congestion. (l) Alveolar epithelial oedema. (m) Normal bronchioles. (n) Exfoliated cells in bronchioles. (o) Mucus and exfoliated cells in bronchioles. All of the bars in the low right corner=100 µm except for that in Figure 1j, which is 50 μm. Two-way ANOVA was used for the viral loads on sacrifice day for different tissues to compare the difference between the experiment and control group (c). The t test was used for the ELISA and pathology score to compare the difference between the experiment and control group (d and e). ***p < 0.001 versus the control group.

Although neutralization titres were detected only in 2 (No. 19256 and No. 19060) of the 4 passively immunized monkeys before virus challenge (Figure 1b), all of the monkeys infused with anti-SARS-CoV-2 IgG showed lower viral loads in nasal swabs, pharyngeal swabs and all of the tissues tested than the monkeys infused with control IgG (Figure 1c), which implied that no ADE of infection was accompanied by nonneutralizing or subneutralizing antibodies after vaccination.

Among all of the inflammatory cytokines (IL-1a, TNF-β, IL-13 and IL-33) and chemokines (IP-10, eotaxin, MCP-1 and IL-8) tested in BALF, some monkeys in the anti-SARS-CoV-2 IgG-infused group showed relatively higher levels of MCP-1, IL-8 and IL-33 than the control group (Figure 1d, S1), although the overall difference between the two groups was not statistically significant. According to the histopathological analysis results of the lung tissues stained with hematoxylin and eosin (H&E), which were observed and blindly scored by two pathologists, the anti-SARS-CoV-2 IgG-infused group and the control group showed a similar pulmonary immunopathology (scored 1–4 for mild, 5–8 for moderate and 9–12 for severe). Alveolar epithelial hyperplasia, alveolar epithelial congestion, mucus and exfoliated cells in bronchioles, and eosinophil infiltration were observed in both anti-SARS-CoV-2 IgG-infused group and control group (Figure 1f-o). Monkey No. 19256 from the anti-SARS-CoV-2 IgG-infused group and monkey No. 19208 from the control group showed relatively more prominent pulmonary immunopathology, with higher histopathology scores of 4.5 and 5, respectively. However, with these two scores both falling into the “moderate” category, the overall difference in immunopathology severity between the two groups was determined to be not statistically significant. (Figure 1e). Besides, no significant differences in immunopathology severity were found in other organs between the two groups.

One way in which SARS-CoV-2 is different from SARS and MERS is the prevalence of a large number of asymptomatic and mild infections. Studies have shown that asymptomatic and mild patients are also capable of producing neutralizing antibodies, the levels of which are positively correlated with disease severity [5]. Recent studies have shown that the neutralizing antibody response attenuates over time, more rapidly with mild infections [13], leading to a greater risk of reinfection with SARS-CoV-2 than with SARS and MERS. In addition, multiple vaccines have now been given emergency use authorization or conditional marketing authorization in multiple countries. Although ADE has not been found in several completed phase III clinical trials, the decline in neutralizing antibody levels within a year of vaccination has been demonstrated in several studies [14,15]. Therefore, whether the nonneutralizing or subneutralizing antibody levels resulting from the decline in neutralizing antibody levels after initial infection or vaccination will result in ADE and promote viral infection or pulmonary immunopathology poses a challenge to the long-term safety of vaccines.

Our study demonstrated that the antibodies purified in vitro from anti-serum of inactivated SARS-CoV-2 vaccination at subneutralizing or even nonneutralizing levels could offer protection from SARS-CoV-2 infection in vivo (Figure 1c). This result is very important for vaccinated people’s primary encounters with trace amounts of virus, which can be eliminated or controlled to prevent proliferative infection. According to the primary clinical phase III trial results of inactivated SARS-CoV-2 vaccines, while the protection rate against coronavirus disease 2019 (COVID-19) was just above 50%, the protection rate against clinically severe cases was nearly 100%, which demonstrated the key role of virus control or elimination upon primary contact in the prevention of severe COVID-19.

On the other hand, the elimination of SARS-CoV-2 by macrophages is reported to be associated with the production of multiple antiviral and proinflammatory cytokines [16], which implies that the elimination of a large number of viruses (10^5^ TCID_50_) administered in rhesus macaque pneumonia models may lead to excessive activation of the immune cascade in lung tissues, which results in pulmonary pathology. Studies of SARS immunization in animal models have yielded widely varying results in terms of protective efficacy and potential ADE, and different vaccine strategies may lead to differences in whether the vaccine enhances the disease. There is a general consensus that inducing higher titres of antibodies as well as antibodies that target the S protein more effectively can reduce the risk of ADE. It is encouraging, however, that the inactivated SARS-CoV-2 vaccine, which targets the full antigen of the virus, provides dose-dependent protection in a variety of animal models without producing evidence of enhanced immunopathological damage [3]. Our study also showed that no statistically significant pulmonary immunopathology was observed in rhesus monkeys passively exposed to either subneutralizing or nonneutralizing antibodies.

In conclusion, our results showed that in the absence of cellular immunity, passive infusion of subneutralizing or nonneutralizing anti-SARS-CoV-2 antibodies could still provide some level of protection against infection upon challenge, and no low-level antibody-enhanced infection was observed. The anti-SARS-CoV-2 IgG-infused group and control group showed similar, mild to moderate pulmonary immunopathology during the acute phase of virus infection, and no evidence of vaccine-related pulmonary immunopathology enhancement was found. There results corresponded with the recent observations that no pulmonary immunology was detected in the preclinical studies of inactivated SARS-CoV-2 vaccines in both murine and NHP pneumonia models or in large clinical trials. Although more evidence is needed to confirm or deny the existence of ADE in SARS-CoV-2 infection and the immune damage caused by it, the available evidence has further supported the safety of the SARS-CoV-2 vaccine.

## Supplementary Material

Supplemental Material

Supplemental Material
