# Supplementary material for: Waning antibodies from inactivated SARS-CoV-2 vaccination offer protection against infection without antibody-enhanced immunopathology in rhesus macaque pneumonia models
Source: Emerg Microbes Infect. 2021 Nov 21;10(1):2194–8. doi: 10.1080/22221751.2021.2002670 (PMC8635581; doi:10.1080/22221751.2021.2002670)
Supplement: Supplemental Material [file TEMI_A_2002670_SM2712.docx]

**Materials and Methods**

1. Animals and ethics statement

A total number of seven male rhesus macaques (age 1.5 years, weight 3-3.5 kg) were used in this study: one was used to produce anti-SARS-CoV-2 IgG (No. 19348); six was used to perform the IgG passive infusion experiment (No. 19208, 19408, 19184, 19146, 19256, 19060). All rhesus macaques were obtained from the Primate Reseach Center of the Institute of Medical Biology, Chinese Academy of Medical Sciences (IMBCAMS), and were housed in cages under Animal Biosafety Level 3 (ABSL-3) laboratory conditions. Sufficient air, natural light and a temperature of 25 °C were maintained to make the monkeys as comfortable as possible; adequate water and food, appropriate veterinarian care were provided daily. Animal protocols were reviewed and approved by the Experimental Animal Management Association of the Institute of Medical Biology (IMB), Chinese Academy of Medical Sciences (CAMS) (approval number: DWSP 202003 005).

2. Virus, cell lines and titration

The KMS-1 strain of SARS-CoV-2 was isolated from respiratory secretions from an adult male patient diagnosed with COVID-19 in Yunnan Hospital of Infectious Diseases in Kunming in January 2020. The virus was later characterized with genomic sequencing and named KMS-1 (GenBank No: MT226610.1). The virus was replicated and titrated in Vero cells under BSL-3 laboratory conditions. The African green monkey kidney Vero cell line (American Type Culture Collection (ATCC), Manassas, VA, USA) was maintained in minimum essential medium (MEM; Invitrogen, Carlsbad, CA, USA) containing 10% foetal bovine serum (FBS; Gibco, Grand Island, NY, USA). Briefly, virus samples were serially diluted 10-fold with serum-free DMEM (Corning, NY, USA), and each dilution (100 µl per well) was added to 8 parallel wells in a 96-well plate. Then, 100 µl of Vero cell suspension at a concentration of 2.5 × 10^5^ cells/ml was added to each well. The plate was then incubated at 37°C with 5% CO_2_ for 7 days before the cytopathic effect (CPE) was observed and assessed with an inverted microscope (Nikon, Tokyo, Japan).

3. Animal vaccination

The inactivated SARS-CoV-2 vaccine was developed by the IMB CAMS as previously described [1]. One male rhesus macaque (age 1.5 years, weight 3.5 kg, No. 19348) was intramuscularly immunized with the vaccine on days 0 and 28. Neutralizing antibody in serum sample was tested 7 days after the booster injection. Once confirming the neutralizing antibody GMT was over 1:128, the monkey was sacrificed, and the whole serum was collected to purify anti-SARS-CoV-2 IgG. For control IgG, rhesus monkeys were immunized with EV-71 inactivated vaccine, which was developed by IMB CAMS using a similar approach with SARS-CoV-2 [2, 3] and approved for marketing previously. Briefly, Enterovirus 71 virus FY-23KB strain was cultured and proliferated in human KMB-17 cells, then inactivated with formalin, purified and emulsified in Al(OH)_3_ adjuvant, constituting the final vaccine. Rhesus macaques were intramuscularly immunized with this vaccine on days 0 and 28. After measuring the neutralizing antibody GMT in the serum, monkeys were sacrificed and whole serum was used to purify IgG.

4. IgG purification

Anti-SARS-CoV-2 IgG was purified from the antiserum of the immunized Rhesus macaques using MabSelect affinity resin (GE Healthcare Bio-Sciences AB, Sweden, cat.: 17519901) according to the manufacturer’s instructions. After the purification, the concentration of the IgG was determined by BCA Protein Assay Kit (Beyotime, Shanghai, cat: P0012) according to the manufacturer’s instructions. Control IgG was previously purified from rhesus macaques immunized with EV-71 inactivated vaccine and stored in the laboratory.

5. Animal study and sample collection

At a neutralizing antibody GMT of 1:128, a whole amount of approximately 1100 mg of IgG was purified from the whole serum of one monkey (No. 19348). Based on this concentration of IgG, a sub-neutralizing or non-neutralizing dose of 10mg/kg of body weight was determined. Six male rhesus macaques (age 1.5 years, weight 3-3.5 kg) were randomly divided into two groups (4 in the anti-SARS-CoV-2 IgG group, No. 19184, 19146, 19256, 19060; 2 in the control IgG group, No. 19208, 19408), and 10 mg/kg body weight of purified anti-SARS-CoV-2 IgG or control IgG were injected intravenously. Three days after IgG injection, all animals were infected with SARS-CoV-2 via bilateral nasal drip at a dose of 10^5^ TCID50. The clinical manifestations of the animals were observed, nasal, pharyngeal swabs and blood samples were collected daily for viral shedding and serum cytokine analysis. Four days after SARS-CoV-2 infection, all animals were sacrificed after anaesthesia, bronchoalveolar lavage fluid and the main tissues and organs were collected for viral load detection and histopathology.

6. Neutralization assay

All experiments were performed under BSL-3 laboratory conditions. A neutralization assay was performed in accordance with standard protocols. Briefly, diluted serum samples (1:4, 1:8, 1:16, 1:32, 1:64, 1:128, and 1:256) were incubated with virus at a titre 100 times higher than the 50% cell culture infectious dose (CCID50)/ml at 37°C for 2 h. The mixture was then added to Vero cells in 96-well plates and incubated at 37°C. The CPE of the virus was observed after 1 week. Neutralization titres under 4 were defined as 1 for calculation.

7. ELISA

IL-1a, TNF-β, IP10, eotaxin, IL-13, IL-33, IL-8, and MCP-1 levels in serum samples and bronchoalveolar lavage fluid samples were determined in triplicate using ELISA kits (eotaxin from Boster Biological Technology Co., Ltd, China; IL-1a, TNF-β, IP10, IL-13, IL-33, IL-8 and MCP-1 from Beijing 4A Biotech Co., Ltd, China) according to the manufacturer’s instructions.

8. qRT-PCR

Viral RNA was extracted from tissue samples using TRIzol reagent (Ambion by Life Technologies, USA). According to the protocol, qRT-PCR was performed using a One Step PrimeScript™ RT-PCR Kit (Perfect Real Time; TaKaRa). The following real-time RT-PCR primer sets were used to measure viral mRNA levels: for N, forward: 5’-GGGGAACTTCTCCTGCTAGAAT-3’, reverse: 5’-CAGACATTTTGCTCTCAAGCTG-3’, and probe: 5’-TTGCTGCTGCTTGACAGATT-3’; and for ORF 1ab, forward: 5’-CCCTGTGGGTTTTACACTTAA-3’, reverse: 5’-ACGATTGTGCATCAGCTGA -3’, and probe: 5’-CCGTCTGCGGTATGTGGAAAGGTTATGG -3’.

9. Histopathology

The main organs and tissues of all monkeys were collected and fixed in 10% neutral buffered formalin, paraffin embedded and sectioned at 4-μm thickness. The sections were then stained using haematoxylin and eosin (H&E) staining kits (Beijing Solarbio Science & Technology Co., Ltd, China) according to the manufacturer’s instructions. Histopathological analysis of the all tissues was conducted by two pathologists by blindly scoring the H&E sections under an inverted microscope (Nikon, Tokyo, Japan). The average estimated severity of the lesions in the lung tissues was determined in at least five areas at 100x as well as 200× magnification and scored as 1 (weak), 2 (moderate) or 3 (severe). The total score (1-4 for mild, 5-8 for moderate and 9-12 for severe) was calculated by adding the scores of the left and right lungs for the superior and inferior lobes of each animal.

10. Statistical analysis

Analysis of data was performed using GraphPad Prism v8.0.2 software (GraphPad Software, CA, USA). The Student’s t-test was used for data analysis, and the differences between the two groups were evaluated by two-way ANOVA.

**References**

1. Che Y, Liu X, Pu Y, et al. Randomized, double-blinded and placebo-controlled phase II trial of an inactivated SARS-CoV-2 vaccine in healthy adults. Clin Infect Dis. 2020 Nov 9. doi: 10.1093/cid/ciaa1703. PubMed PMID: 33165503; PubMed Central PMCID: PMCPMC7717222.

2. Dong C, Wang J, Liu L, et al. Optimized development of a candidate strain of inactivated EV71 vaccine and analysis of its immunogenicity in rhesus monkeys. Hum Vaccin. 2010 Dec;6(12):1028-37. doi: 10.4161/hv.6.12.12982. PubMed PMID: 21150270.

3. Dong C, Liu L, Zhao H, et al. Immunoprotection elicited by an enterovirus type 71 experimental inactivated vaccine in mice and rhesus monkeys. Vaccine. 2011 Aug 26;29(37):6269-75. doi: 10.1016/j.vaccine.2011.06.044. PubMed PMID: 21722686.
