# Supplementary figures and images for: Waning antibodies from inactivated SARS-CoV-2 vaccination offer protection against infection without antibody-enhanced immunopathology in rhesus macaque pneumonia models
Source: Emerg Microbes Infect. 2021 Nov 21;10(1):2194–8. doi: 10.1080/22221751.2021.2002670 (PMC8635581; doi:10.1080/22221751.2021.2002670)

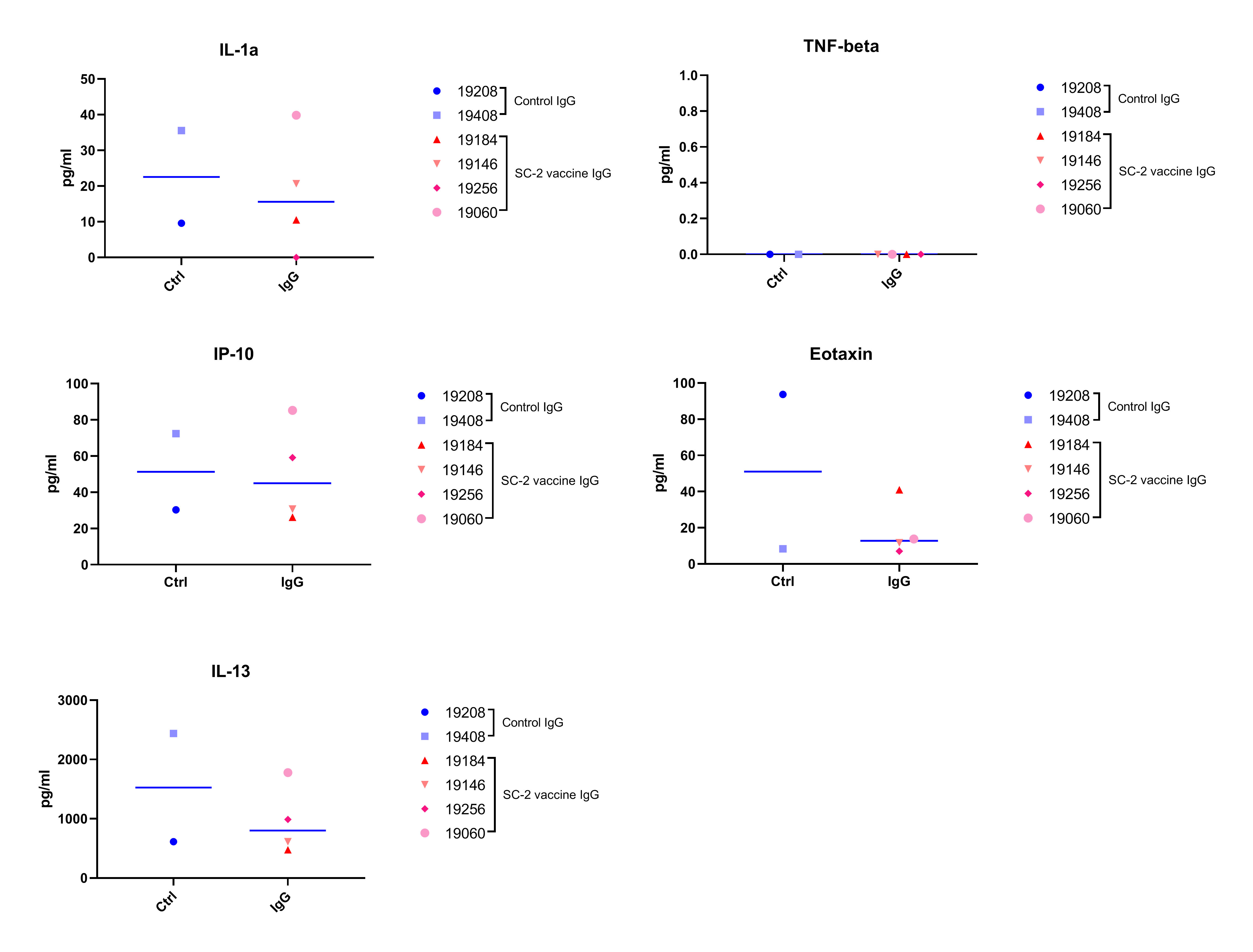

Supplement: Supplemental Material [file TEMI_A_2002670_SM2693.jpg]
